# The relationship of lymphatic vessel density, lymphovascular invasion, and lymph node metastasis in breast cancer: a systematic review and meta-analysis

**DOI:** 10.18632/oncotarget.13752

**Published:** 2016-12-01

**Authors:** Song Zhang, Dong Zhang, Shanhong Yi, Mingfu Gong, Caibao Lu, Yuanqing Cai, Xuefeng Tang, Liguang Zou

**Affiliations:** ^1^ Department of Radiology, Xinqiao Hospital, Third Military Medical University, Chongqing 400037, China; ^2^ Department of Urology, Xinqiao Hospital, Third Military Medical University, Chongqing 400037, China; ^3^ Department of Nephrology, Xinqiao Hospital, Third Military Medical University, Chongqing 400037, China; ^4^ Department of Pathology, Xinqiao Hospital, Third Military Medical University, Chongqing 400037, China

**Keywords:** lymphatic microvessel density, lymphovascular invasion, lymph node metastasis, breast cancer, meta-analysis

## Abstract

Lymph node status is one of the key parameters used for determining the stage of breast cancer progression. The relationship of lymphatic vessel density (LVD), lymphovascular invasion (LVI), and lymph node metastasis (LNM) has not been clearly demonstrated yet. Databases of PubMed, Embase, and Web of Science were searched from inception up to 25 May 2016. Spearman correlation coefficient (*r*) and 95% confidence interval (CI) were used to determine the relationship within each group. Based on pre-established inclusion criteria, 28 studies involving 2920 breast cancer patients were included in this study. The *r* values of LVD-LVI, LVD-LNM, and LVI-LNM were 0.45 (95% CI: 0.31 to 0.57), 0.32 (95% CI: 0.23 to 0.40), and 0.24 (95% CI: 0.19 to 0.28), respectively. Compared with intratumoral LVD, peritumoral LVD showed more robust correlation with LVI (*r* = 0.53, 95% CI: 0.27 to 0.72) and LNM (*r* = 0.33, 95% CI: 0.18 to 0.46). The patients in LNM positive group presented with higher LVI detection rate of 45.85%, while in LNM negative group with detection rate of 23.85%. The results describe a triangle relationship between LVD, LVI, and LNM in breast cancer. Both LVD and LVI are indicated to be valuable predictors of LNM occurrence. Compared with intratumoral lymphatic vessels, peritumoral lymphatics might be the main disseminate route for breast tumor cells.

## INTRODUCTION

Cancer metastasis is the leading cause of mortality in patients diagnosed with breast cancer and other malignant tumors [1]. Lymph node status is commonly used to identify a patient's prognosis, tumor stage, and treatment modality [2]. Patients without lymph node metastasis (LNM) have a favorable prognosis, while with more than six positive axillary lymph nodes have a higher risk of distant metastasis [3]. The progress of lymphatic metastasis is thought to involve the proliferation of lymphatic vessels (lymphangiogenesis), lymphovascular invasion, and LNM step by step [1]. However, the mechanism leading to tumor cells spread via lymphatic vessels (lymphovascular invasion, LVI) to the regional and distant lymph nodes has not been clearly demonstrated [4].

Lymphatic vessel was thought to play a passive role in tumor metastasis, due to the absence of reliable molecular markers to distinguish lymphatic vessels and the lack of identified growth factors for the lymphatic system. During the last two decades, substantial progress within the field has rapidly lead to the recognition of the lymphatic system as an active player involved in lots of malignant tumors [5]. Lymphatic vessels not only provide an entrance for tumor cells to penetrate in [6], but also make several key contributions to tumor metastasis, such as the provision of a niche for cancer stem cells and the modulation of antitumor immune responses [4]. It is known that tumor angiogenesis and its indicator blood vessel denstiy are closely associated with the clinicopathological outcomes in breast cancer [7]. However, the clinical role of lymphangiogenesis and its indicator lymph vessel density (LVD) is needed to be further investigated [8, 9].

Despite lymphatic metastasis is thought to facilitate tumor cell dissemination in breast cancer [10], the possible relationship of LVD, LVI, and LNM remains ambiguous. Questions can be listed as follows: (1) do lymphangiogenesis facilitate the occurrence of LVI and LNM in breast cancer? (2) regarding LVI detection rate, is there any difference between LNM negative group and LNM positive group? (3) whether lymphangiogenesis is previous to LVI, or it is promoted by the chemokines secreted by tumor cells penetrated into lymphatic vessels? The answers to these questions are important to understand whether tumor-induced lymphangiogenesis is a potential target for the inhibition of distant metastasis, as well as whether high LVD and LVI presence are valuable factors to predict LNM occurrence in breast cancer. With the accumulating evidence, we conducted a systematic review and meta-analysis to estimate the relationship of LVD, LVI, and LNM in breast cancer to provide insights of the above issues.

## RESULTS

### Study selection process

**Figure 1:**
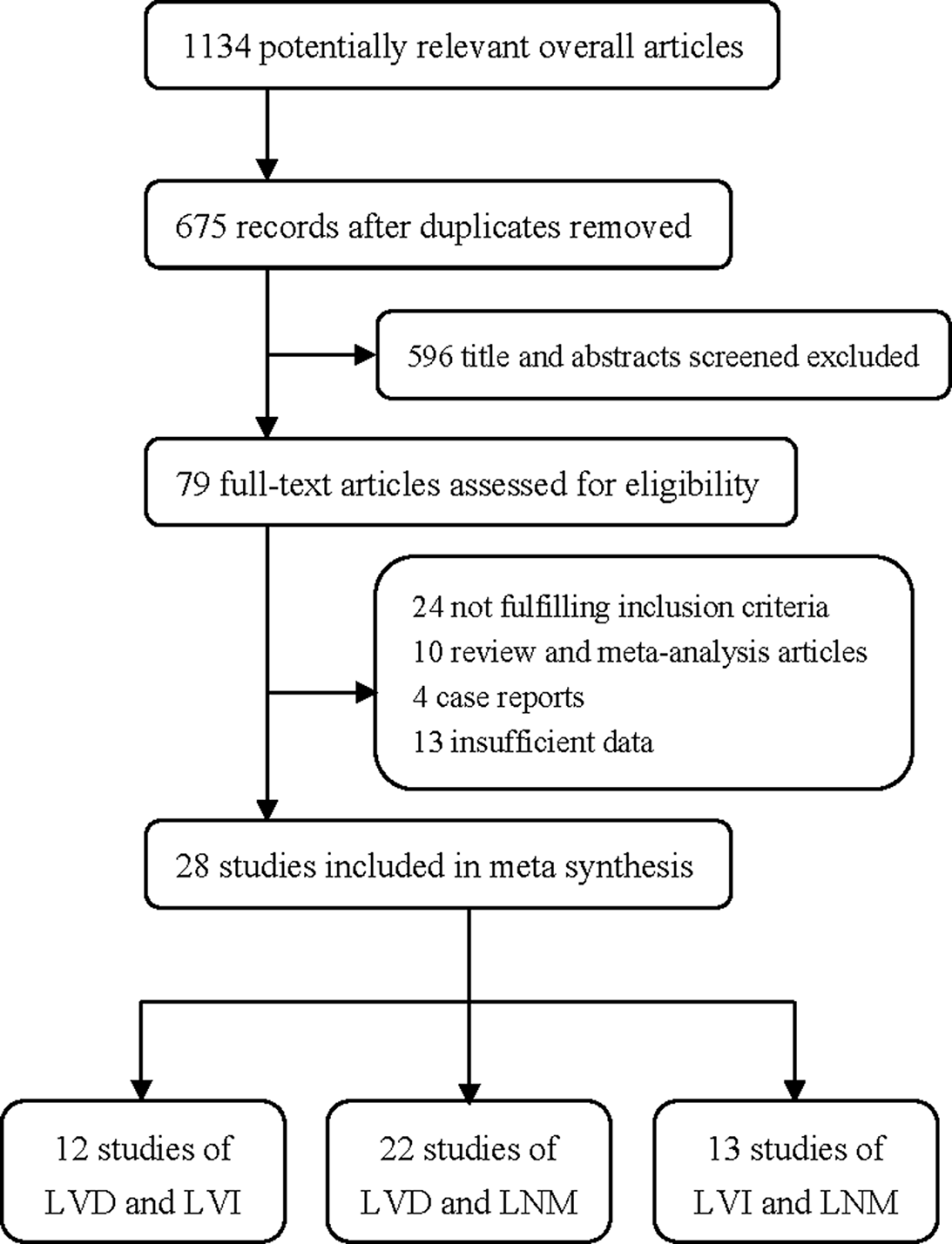
Flowchart of study selection

The literature search result is shown in the flowchart of Figure 1. We initially searched 1134 potential studies from the databases. After removing the duplicated and irrelevant publications, 79 full-text publications were left over to assess the eligibility. Fifty-one papers were excluded due to not fulfill the inclusion criteria, inappropriate publication types, or insufficient data. Finally, 28 articles were included in the analysis [11–38].

### Characteristics of the included studies

The results of all included 28 studies were exhibited in Tables 1–3. The sample size of each study ranged from 29 to 374 patients, and the publication year of them ranged from 2000 to 2016. A total of 2920 breast cancer patients were adopted in this study. All patients were performed surgical treatments, and IHC staining with D2–40, podoplanin, LYVE-1, and VEGFR-3 antibodies. LVD was determined by counting the number of lymphatic vessels per area under a microscope. LVI was defined as the presence of tumor cells in lymphatic vessels. Lymph nodes, either sentinel lymph nodes or non-sentinel lymph nodes, were taken into account to determine the occurrence of LNM.

**Table 1:** Main characteristics and results of the studies evaluating LVD and LVI

Author, Year, Country	No. of patients	Age	Tumor type	Antibody (dilution)	LVD of LVI^-^ (No.)	LVD of LVI^+^ (No.)	Area	*r* (95% CI)
Abe, 2016, Japan [11]	91	54 (30–81)^a^	invasive ductal BC	D2-40 (1:100)	4.42 ± 3.97 (53)	11.16 ± 5.40 (38)	total	0.58 (0.41, 0.72)
Widodo, 2013, Indonesia [36]	48	53.0 (34–75)^a^	breast cancer	D2-40 (1:75)	6.00 ± 4.06 (13)	9.62 ± 3.17 (35)	peritumoral	0.43 (0.16, 0.63)
Ding, 2012, China [16]	75	52.1 (42–63)^a^	ductal invasive BC and Paget disease	D2-40 (NG)	11.11 ± 6.76 (39)	18.12 ± 9.06 (36)	peritumoral	0.40 (0.19, 0.58)
Kandemir, 2012, Turkey [22]	69	54.8 (39–85)^a^	ductal invasive BC	D2-40 (1:50)	15.41 ± 0.1 (26) 55.29 ± 1.6 (26)	22.13 ± 0.9 (43) 91.02 ± 2.5 (43)	intratumoral peritumoral	0.98 (0.90, 0.99) 0.99 (0.93, 1.00)
Zhao, 2012, China [38]	73	53.8 (29–75)^a^	ductal invasive BC	D2-40 (1:25)	5.57 ± 2.11 (48) 8.04 ± 2.89 (48)	5.29 ± 1.96 (25) 10.19 ± 3.61 (25)	intratumoral peritumoral	–0.06 (–0.28, 0.16) 0.31 (0.08, 0.50)
Lee, 2010, Korea [25]	46	47.9 ± 2.5^c^	microinvasive ductal BC	D2-40 (1:130)	5.32 ± 1.97 (39)	6.00 ± 2.56 (7)	total	0.12 (–0.17, 0.38)
El-Gendi, 2009, Egypt [17]	40	51.5 (27–92)^b^	invasive BC	D2-40 (1:50)	7.5 (0.0–45.0)b (29)	6.7 (3.3-12.0)b (11)	total	–0.01 (–0.31, 0.28)
Mohammed, 2009, UK [27]	177	57 (32–70)^b^	invasive BC	D2-40 (1:100)	L (109), H (14)	L (13), H (41)	total	0.64 (0.51, 0.74)
El-Gohary, 2008, USA [18]	48	64 (27–89)^a^	invasive BC	D2-40 (1:50)	NG (30)	NG (18)	intratumoral peritumoral	0.54 (0.30, 0.72) 0.54 (0.30, 0.72)
Kato, 2005, UK [24]	67	49 (30–86)^b^	primary BC	LYVE-1 (1:600)	5.9 ± 3.8 (42)	6.8 ± 4.8 (25)	total	0.10 (–0.13, 0.33)
Nakamura, 2005, Japan [29]	113	51 (24–87)^b^	invasive BC	podoplanin (1:200)	6.54 ± 4.92 (56)	13.63 ± 7.82 (57)	total	0.48 (0.31, 0.61)
Schoppmann, 2004, Austria [31]	374	57.6^b^	invasive BC	podoplanin (1:200)	8.3 ± 4.2 (269)	12 ± 4.2 (105)	total	0.37 (0.27, 0.46)

^a^ mean (range); ^b^ median (range); ^c^ mean ± SD; BC, breast cancer; H, high LVD; L, low LVD; NG, not given.

**Table 2:** Main characteristics and results of the studies evaluating LVD and LNM

Author, Year, Country	No. of patients	Age	Tumor type	Antibody dilution	LVD of LNM^-^ (No.)	LVD of LNM^+^ (No.)	Area	*r* (95% CI)
Abe, 2016, Japan [11]	91	54 (30–81)^a^	invasive ductal BC	D2-40 (1:100)	5.56 ± 4.48 (38)	8.44 ± 6.16 (53)	total	0.25 (0.05, 0.43)
Zhang, 2015, China [37]	106	34 (26–35)^a^ (51) 50 (40–67)^a^ (56)	invasive ductal BC	LYVE-1 (NG)	L (25), H (37)	L (19), H (25)	total	−0.03 (−0.22, 0.16)
Widodo, 2013, Indonesia [36]	48	53.0 (34–75)^a^	breast cancer	D2-40 (1:75)	7.88 ± 3.05 (18)	9.09 ± 4.17 (30)	peritumoral	0.15 (−0.13, 0.41)
Ding, 2012, China [16]	75	52.1 (42–63)^a^	ductal invasive BC and Paget disease	D2-40 (NG)	9.95 ± 6.46 (43)	15.36 ± 8.36 (32)	peritumoral	0.34 (0.12, 0.53)
Kandemir, 2012, Turkey [22]	69	54.8 (39–85)^a^	ductal invasive BC	D2-40 (1:50)	7.4 ± 1.3 (26) 52.5 ± 11.5 (26)	14.8 ± 5.1 (43) 75.1 ± 12.3 (43)	intratumoral peritumoral	0.66 (0.47, 0.79) 0.67 (0.49, 0.80)
Zhao, 2012, China [38]	73	53.8 (29–75)^a^	ductal invasive BC	D2-40 (1:25)	5.58 ± 1.92 (34) 7.57 ± 3.10 (34)	5.38 ± 2.15 (39) 9.82 ± 3.13 (39)	intratumoral peritumoral	−0.05 (−0.27, 0.18) 0.34 (0.12, 0.53)
Lee, 2010, Korea [25]	46	47.9 ± 2.5^c^	microinvasive ductal BC	D2-40 (1:130)	5.14 ± 2.07 (37)	6.59 ± 1.61 (9)	total	0.28 (−0.01, 0.52)
Britto, 2009, Brazil [14]	92	55 (32–77)^b^	BC	D2-40 (1:50)	7 (1–20)^b^ (61)	8 (0-22)b (31)	total	0.09 (−0.11, 0.29)
El-Gendi, 2009, Egypt [17]	40	51.5 (27–92)^b^	invasive BC	D2-40 (1:50)	6.75 (0–15.7)^b^ (14)	8.85 (0-45)b (24)	total	0.39 (0.09, 0.63)
Mohammed, 2009, UK [27]	177	57 (32–70)^b^	invasive BC	D2-40 (1:100)	L (104), H (21) L (81), H (44) L (77), H (48)	L (18), H (34) L (23), H (29) L (13), H (39)	total intratumoral peritumoral	0.48 (0.34, 0.60) 0.19 (0.04, 0.33) 0.33 (0.18, 0.47)
El-Gohary, 2008, USA [18]	48	64 (27–89)^a^	invasive BC	D2-40 (1:50)	NG (24) NG (24)	NG (24) NG (24)	intratumoral peritumoral	0.49 (0.24, 0.68) 0.35 (0.07, 0.57)
Gu, 2008, China [19]	61	57.59 (29–90)^a^	BC	podoplanin (1:25)	4.24 ± 3.01 (29)	8.31 ± 3.38 (32)	total	0.54 (0.31, 0.70)
Mylona, 2007, Greece [28]	109	56.89 (25–86)^a^	invasive BC	D2-40 (1:20)	9.5 (3−23)^b^ (44)	10 (4-30)b (65)	total	0.04 (−0.14, 0.22)
van der Schaft, 2007, Netherlands [34]	121	61.4 ± 12.2c	ductal invasive BC	podoplanin (NG)	0.04 ± 1.44 (70) 4.74 ± 3.80 (70)	0.29 ± 1.06 (51) 4.59 ± 4.29 (51)	intratumoral peritumoral	0.10 (−0.08, 0.27) −0.02 (−0.19, 0.16)
van Iterson, 2007, Finland [35]	95	NG	lobular invasive BC	LYVE-1 (1:300)	3.2 ± 1.5 (31)	4.6 ± 1.6 (64)	peritumoral	0.39 (0.20, 0.55)
Guo, 2006, China [20]	51	52.3 (38–67)^a^	invasive BC	VEGFR-3 (NG)	19.49 ± 2.80 (10)	29.24 ± 3.44 (41)	total	0.76 (0.57, 0.87)
Choi, 2005, USA [15]	29	66 (34–91)^b^	invasive BC	D2-40 (1:5)	NG (15)	NG (14)	total	0.36 (−0.01, 0.64)
Kato, 2005, UK [24]	67	49 (30–86)^b^	primary BC	LYVE-1 (1:600)	6.4 ± 4.1 (43)	6.3 ± 4.5 (20)	total	−0.01 (−0.25, 0.23)
Nakamura, 2005, Japan [29]	113	51 (24–87)^b^	invasive BC	podoplanin (1:200)	5.74 ± 3.69 (57)	14.9 ± 7.54 (56)	total	0.61 (0.46, 0.73)
Bono, 2004, UK [12]	180	57 (34–89)^b^	invasive ductal BC	LYVE-1 (NG)	L (61), H (46)	L (32), H (41)	total	0.13 (−0.02, 0.27)
Schoppmann, 2004, Austria [31]	374	57.6 (median)	invasive BC	podoplanin (1:200)	8.9 ± 4.2 (212)	9.8 ± 4.9 (162)	total	0.10 (0.00, 0.20)
Nathanson, 2000, USA [30]	60	53 (28–81)^b^	stage II BC	VEGFR-3 (NG)	4 ± 4.16 (27)	16 ± 8.04 (33)	total	0.67 (0.48, 0.81)

^a ^mean (range); ^b ^median (range); ^c ^mean ± SD; BC, breast cancer; H, high LVD; L, low LVD; NG, not given.

**Table 3:** Main characteristics and results of the studies evaluating LVI and LNM

Author, Year, Country	No. of patients	Age	Tumor type	Antibody dilution	LVI of LNM^-^(No.)	LVI of LNM^+^(No.)	*r* (95% CI)
Kanngurn, 2013, Thailand [23]	122	52 (29–86)^b^	invasive primary BC	D2-40 (1:200)	N (68), P (11)	N (21), P (18)	0.35 (0.17, 0.51)
Widodo, 2013, Indonesia [36]	48	53.0 (34–75)^a^	breast cancer	D2-40 (1:75)	N (6), P (12)	N (7), P (23)	0.11 (−0.17, 0.38)
Kandemir, 2012, Turkey [22]	69	54.8 (39–85)^a^	ductal invasive BC	D2-40 (1:50)	0.06 ± 0.05 (15)	0.19 ± 0.21 (28)	0.32 (0.03, 0.57)
Lee, 2010, Korea [25]	46	47.9 ± 2.5^c^	microinvasive ductal BC	D2-40 (1:130)	N (33), P (4)	N (6), P (3)	0.25 (−0.06, 0.51)
Britto, 2009, Brazil [14]	92	55 (32–77)^b^	BC	D2-40 (1:50)	N (44), P (17)	N (21), P (10)	0.05 (−0.16, 0.25)
Braun, 2008, Germany [13]	254	57 (28–85)^b^	primary invasive BC	D2-40 (1:50)	N (114), P (20)	N (49), P (44)	0.35 (0.22, 0.47)
El-Gohary, 2008, USA [18]	48	64 (27–89)^a^	invasive BC	D2-40 (1:50)	NG (24)	NG (24)	0.52 (0.27, 0.70)
Marinho, 2008, Brazil [26]	123	52 (27–88)^b^	invasive BC	D2-40 (1:100)	N (32), P (9)	N (56), P (26)	0.10 (−0.08, 0.27)
Ito, 2007, Japan [21]	69	52.1 (27–80)^a^	invasive BC	D2-40 (1:200)	N (37), P (7)	N (16), P (9)	0.23 (−0.01, 0.45)
Tezuka, 2007, Japan [32]	132	55.9 (31–84)^b^	invasive BC	D2-40 (NG)	N (42), P (21)	N (35), P (34)	0.16 (−0.01, 0.32)
van den Eynden, 2006, Belgium [33]	95	60.5 (33.5–86.1)^a^	invasive BC	D2-40 (1:100)	N (19), P (33)	N (9), P (33)	0.16 (−0.04, 0.36)
Kato, 2005, UK [24]	67	49 (30–86)^b^	primary BC	LYVE-1 (1:600)	N (31), P (12)	N (8), P (12)	0.31 (0.06, 0.52)
Schoppmann, 2004, Austria [31]	374	57.6 (median age)	invasive BC	podoplanin (1:200)	N (171), P (41)	N (98), P (64)	0.22 (0.12, 0.32)

^a^ mean (range); ^b^ median (range); ^c^ mean ± SD; BC, breast cancer; N, LVI negative; P, LVI positive; NG, not given.

### Data analysis

**Figure 2:**
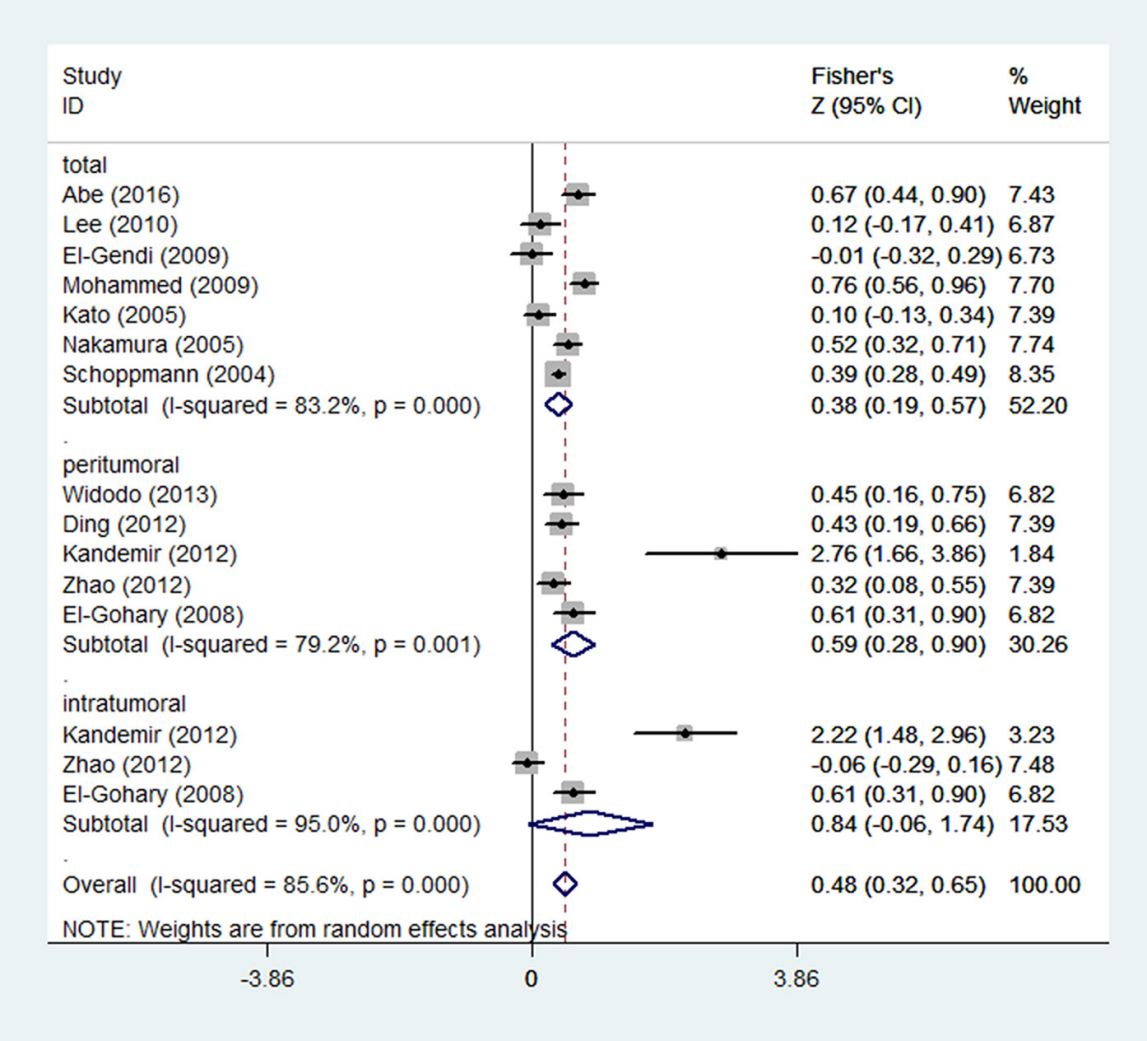
Forest plot of the Fisher's Z values for the correlation between LVD and LVI in breast cancer

A total of 1221 patients involved in 12 studies (Table 1), which provided sufficient data to determine the relationship between LVD and LVI. According to the detection area of lymphatic vessels, the included 12 studies were divided into three subgroups as follows: total LVD (without distinguishing peritumoral and intratumoral) (*n* = 7) [11, 17, 24, 25, 27, 29, 31], peritumoral LVD (*n* = 5) [16, 18, 22, 36, 38], and intratumoral LVD (*n* = 3) [18, 22, 38]. The main outcomes are summarized in Figure 2. The pooled Fisher's Z values of total LVD, peritumoral LVD, intratumoral LVD, overall LVD were 0.38 (95% CI: 0.19 to 0.57, *I*^2^ = 83.2%, *P* = 0.000), 0.59 (95% CI: 0.28 to 0.90, *I*^2^ = 79.2%, *P* = 0.001), 0.84 (95% CI: −0.06 to 1.74, *I*^2^ = 95%, *P* = 0.000), 0.48 (95% CI: 0.32 to 0.65, *I*^2^ = 85.6%, *P* = 0.000), respectively. The pooled Fisher's Z values were converted back to *r* values. Both total LVD (*r* = 0.36, 95% CI: 0.19 to 0.52) and overall LVD (*r* = 0.45, 95% CI: 0.31 to 0.57) were moderately correlated with LVI, while peritumoral LVD (*r* = 0.53, 95% CI: 0.27 to 0.72) and intratumoral LVD (*r* = 0.69, 95% CI: −0.06 to 0.94) showed strong correlation with LVI (Figure 2, Table 4). However, only three sets of intratumoral LVD data were acquired. Significant evidence of heterogeneity was noted among these studies (*P* = 0.05, *I*^2^ = 95%).

**Table 4:** Pooled *Z* values, pooled *r* values, Begg's and Egger's results of each correlation group

Relation group	Pooled *Z* value(95% CI)	Pooled *r* value(95% CI)	Begg's test(*P* value)	Egger's test(*P* value)
total LVD-LVI (*n* = 7)	0.38 (0.19, 0.57)	0.36 (0.19, 0.52)	0.356	0.678
peritumoral LVD-LVI (*n*= 5)	0.59 (0.28, 0.90)	0.53 (0.27, 0.72)	0.068	0.005
intratumoral LVD-LVI (*n*= 3)	0.84 (−0.06, 1.74)	0.69 (−0.06, 0.94)	0.296	0.225
overall LVD-LVI (*n*= 15)	0.48 (0.32, 0.65)	0.45 (0.31, 0.57)	0.700	0.000
total LVD-LNM (*n* = 15)	0.33 (0.19, 0.48)	0.32 (0.19, 0.45)	0.023	0.038
peritumoral LVD-LNM (*n* = 8)	0.34 (0.18, 0.50)	0.33 (0.18, 0.46)	0.711	0.321
intratumoral LVD-LNM (*n* = 5)	0.30 (0.04, 0.55)	0.29 (0.04, 0.50)	0.806	0.283
overall LVD-LNM (*n* = 28)	0.33 (0.23, 0.42)	0.32 (0.23, 0.40)	0.047	0.005
LVI-LNM (*n*= 13)	0.24 (0.19, 0.29)	0.24 (0.19, 0.28)	0.428	0.736

**Figure 3:**
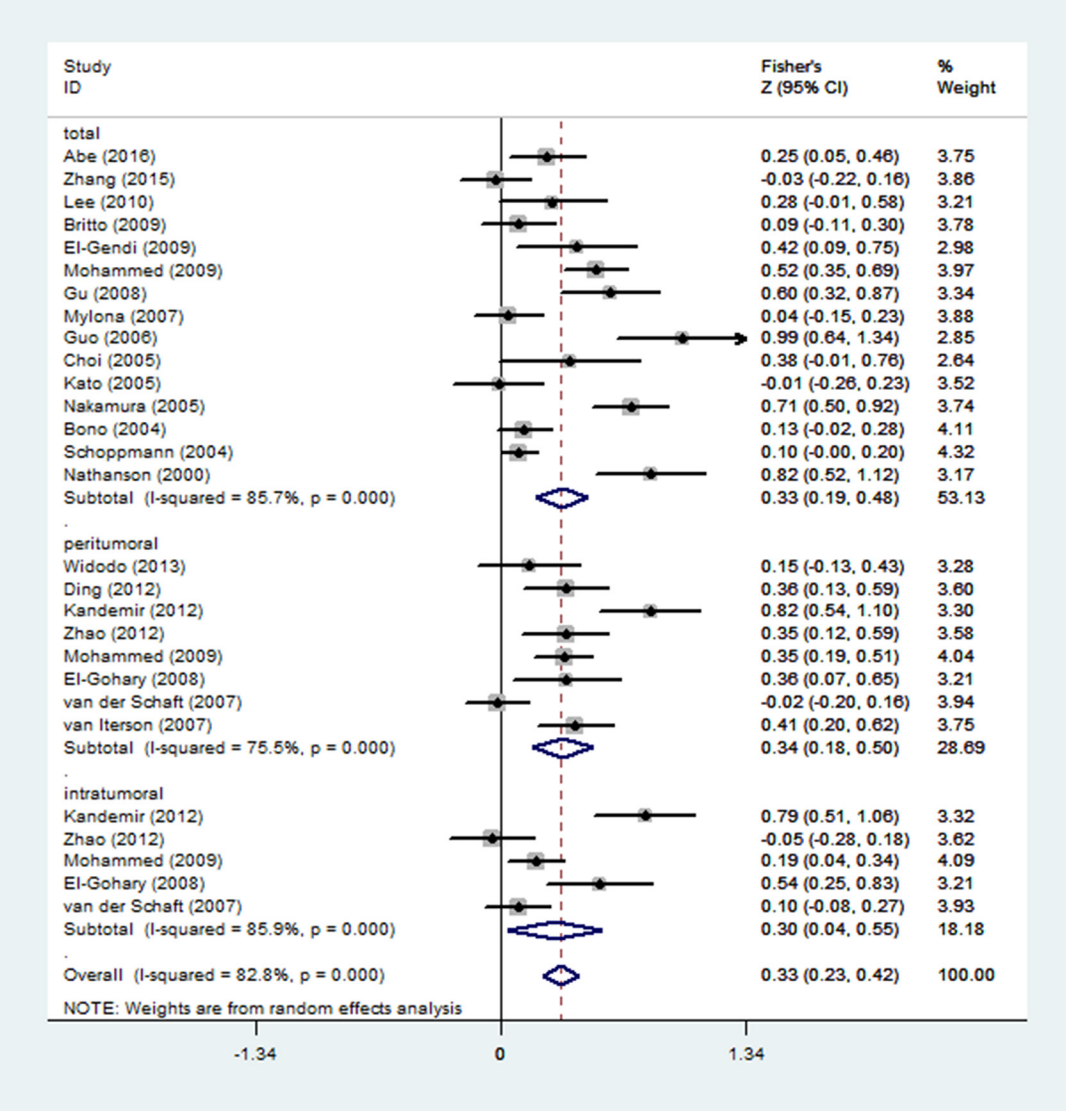
Forest plot of the Fisher's Z values for the correlation between LVD and LNM in breast cancer

Twenty-two studies involving 2125 patients were included in the assessment of the correlation between LVD and LNM (Table 2). According to the detection area of lymphatic vessels, the studies were also divided into three subgroups of total LVD (*n* = 15) [11, 12, 14, 15, 17, 19, 20, 24, 25, 27–31, 37], peritumoral LVD (*n* = 8) [16, 18, 22, 27, 34–36, 38], and intratumoral LVD (*n* = 5) [18, 22, 27, 34, 38]. The pooled Fisher's *Z* values were show in Figure 3, corresponding to the *r* values of total LVD (*r* = 0.32, 95% CI: 0.19 to 0.45), peritumoral LVD (*r* = 0.33, 95% CI: 0.18 to 0.46), intratumoral LVD (*r* = 0.29, 95% CI: 0.04 to 0.50), and overall LVD (*r* = 0.32, 95% CI: 0.23 to 0.40), respectively (Figure 3, Table 4).

**Figure 4:**
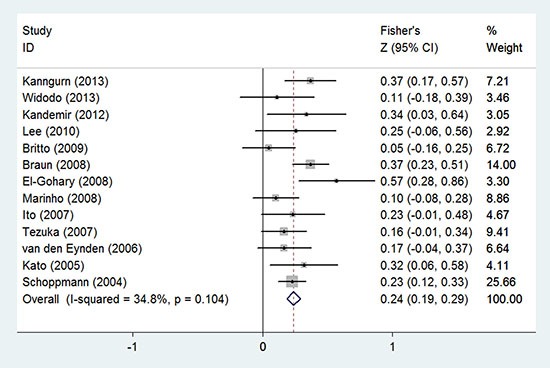
Forest plot of the Fisher's Z values for the correlation between LVI and LNM in breast cancer

Among the included 13 studies, which provided sufficient data to evaluate the relationship between LVI and LNM, eleven of them [13, 14, 21, 23–26, 31–33, 36] described the detailed number of LVI occurrence within LNM negative group and LNM positive group. In LNM negative group (*n* = 784), 187 (23.85%) patients presented with LVI; while in LNM positive group (*n* = 602), 276 (45.85%) patients presented with LVI. To evaluate the correlation of LVI and LNM, all aquired data were used to obtain the *r* values, and then were transformed to the Fisher's Z values. The pooled Fisher's Z value was 0.24 (95% CI: 0.19 to 0.29, *I*^2^ = 34.8%, *P* = 0.104, Figure 4), and its corresponding *r* value was 0.24 (95% CI: 0.19 to 0.28).

### Sensitivity analysis and publication bias

In order to assess the stability of the results, sensitivity analyses were independently performed in the groups of LVD-LVI, LVD-LNM, and LVI-LNM. By removing individual studies in turn, sensitivity analyses demonstrated no disproportionate pooled estimates, indicating a statistically robust result of the analysis (Figure S1–S3 in Supplementary file 1). Begg's tests and the funnel plots of the *Z* value against the standard error of Z value showed substantial asymmetry (Figure S4–S6 in Supplementary file 1). The results of Begg's and Egger's tests are displayed in Table 4.

## DISCUSSION

**Figure 5:**
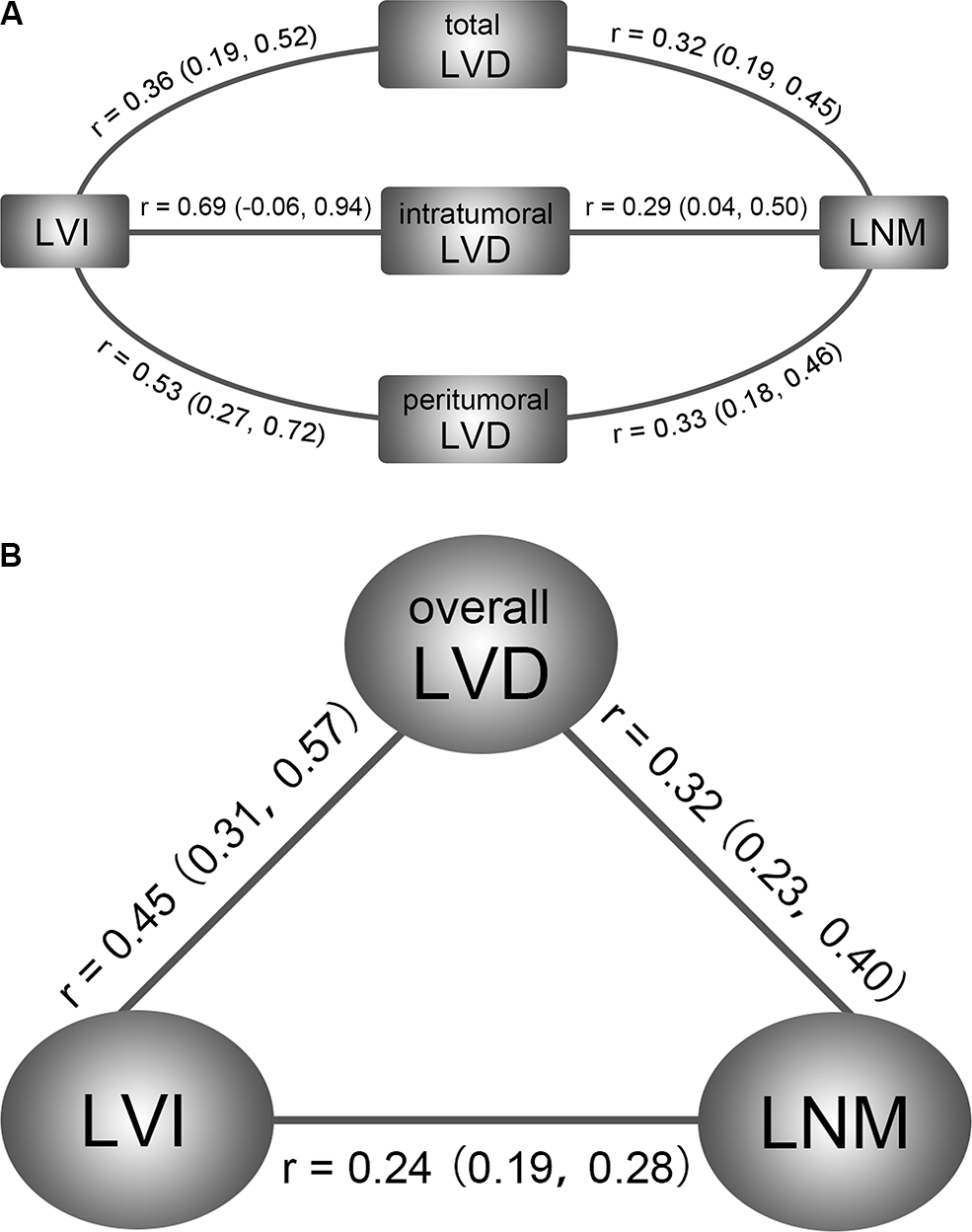
Correlation models of the meta-analysis study **(A)** Relationships of LVD-LVI and LVD-LNM in the subgroups of general LVD, intratumoral LVD, and peritumoral LVD; **(B)** Triangle relationship model of overall LVD, LVI and LNM in breast cancer.

The current meta-analysis included 28 studies with an overall population of 2920 breast cancer patients. Our study reveals the triangle relationship of LVD, LVI, and LNM in breast cancer (Figure 5). Peritumoral LVD shows the most robust correlation with LVI and LNM, while intratumoral LVD and total LVD presents with a relatively weak correlation (Figure 5). Patients in LNM positive group shows higher LVI detection rate than that of LNM negative group. The results demonstrate that both LVD and LVI are valuable predictors of the LNM occurrence in breast cancer. However, the evidence of heterogeneity was observed across the studies, which needed to be further investigated.

Heterogeneity is a potential problem to interpret the meta-analysis results. Significant heterogeneities between-studies were presented. The detailed LVD values and LVI detection rate differentiate notably among these studies. The variation might be caused by patient sources, staining techniques, antibody categories and antibody dilutions. In addition, different counting methods of lymphatic vessel density, by using different hotspots (three [19], four [34], and five [11]), magnification field (100× [27], 200× [11], 400× [19]), and measuring unit (vessels/mm^2^ [11], vessels/field [31]), are also accounted for the variation of results. Furthermore, the cutoff value to divide LVD and LVI as low and high is a crucial factor that cannot be ignored. Because the asset values of LVD and LVI is not normal distribution, most of the included studies chose the median value as the cutoff value, a few of them took the mean or actual value as the cutoff value. Therefore, studies with more standardized and stricter design are required for the assessment of lymphatic vessel density.

Over decades, lymphatic vessels have been described as a passive participant in metastasis and regarded as only a transportation channel of tumor cells. It is still uncertain whether a high LVD is a necessary condition for metastasis [39]. Zhang et al. demonstrated that tumor invasion, but not lymphangiogenesis, was correlated with LNM and unfavorable prognosis in young breast cancer patients [37]. Other studies found that the LVD in LVI/LNM negative group even higher than that of LVI/LNM positive group in primary breast cancer [17, 24, 38]. Nonetheless, most of the included studies showed a positive correlation between a high LVD and the presence of LVI/LNM. The meta-analysis results showed positive correlations between LVD and LVI (*r* = 0.45, 95% CI: 0.31 to 0.57), LVD and LNM (*r* = 0.32, 95% CI: 0.23 to 0.40). It indicates that lymphangiogenesis may facilitate the interaction between tumor cells and lymphatic vessels, thereby increasing the probability of tumor cells invasion and distant metastasis. In addition, lymphatic vessel may provide a safe route for cancer cell dissemination, due to the discontinuous structure of the lymphatic basement membrane, a miniature shear stress and a high concentration of hyaluronic acid [39].

The presence of intratumoral lymphatic vessels is a hotly debated issue in malignant solid tumors, particularly in breast cancer [12, 40, 41]. Previous studies reported that solid tumors did not have intratumoral lymphatic vessels [42], because of the increasing interstitial pressure induced by the proliferating tumor cells [43]. With the application of specific lymphatic vessel markers, many studies have demonstrated the presence of intratumoral lymphatic vessels [27, 44]. Moreover, intratumoral lymphatic vessels are indicated to be functional, because tumor cells can be found within the vessels [45]. Another interesting issue is to what extent intratumoral lymphatic vessels and peritumoral lymphatic vessels participate in tumor cells dissemination. Our study shows that high peritumoral LVD strongly correlates with LVI (*r* = 0.53, 95% CI: 0.27 to 0.72), while high intratumoral LVD moderately correlates with LVI (*r* = 0.33, 95% CI: 0.18 to 0.46). The result suggests that peritumoral lymphatic vessels play a more important role on metastatic dissemination in breast cancer.

The detection of LVI was mainly assessed by H&E staining method, due to the deficiency of specific markers of lymphatic vessels [46, 47]. One major challenge of this method is to distinguish LVI from the retraction artifacts caused by tissue handling and fixation. A previous meta-analysis study has summarized the data on the presence of LVI in breast cancer [48]. The results show that the detection rate of LVI widely ranges from 10% to 49% of H&E staining method, while the range is narrower (from 21% to 42%) by using IHC staining method [48]. It indicates that IHC staining method should be more reliable to identify the presence of LVI. Therefore, 13 studies, by using IHC instead of H&E, were included to investigate the relationship between LVI and LNM. Eleven of them reported the detailed LVI presence in LNM negative group (overall detection rate of 23.85%) and positive group (overall detection rate of 45.85%). The presence of LVI shows a weak correlation with the occurrence of LNM in breast cancer (*r* = 0.24, 95% CI: 0.19 to 0.28).

The current meta-analysis study has some strengths. It is the first study to systematically discuss the triangle relationship of LVD, LVI, and LNM in breast cancer. The included 28 studies and 2290 participants significantly enhanced the statistical power and provided more reliable results. However, some limitations should not be ignored. First, all included studies were observational studies, the sample sizes were relatively small, and several studies were excluded due to lack of sufficient data to determine the correlation coefficients. Thus, selection bias and recall bias are inevitable. Second, most of the included studies investigated lymphatic vessels without distinguishing intratumoral and peritumoral lymphatic vessels, which would confound the final results. Finally, as described above, significant between-studies heterogeneities were presented. Therefore, the standardization of LVD and LVI counting method needs to be established for the future study.

## MATERIALS AND METHODS

### Literature search

A systematic search of the PubMed, Embase and Web of Science databases was performed to identify all relevant articles published up to 25 May 2016. The following Medical Subject Heading (MeSH) terms or keywords were used: “breast cancer OR breast carcinoma OR breast neoplasms” AND “lymphatic vessel density OR lymphatic microvessel density OR LVD OR LMVD OR lymphangiogenesis OR lymphovascular invasion OR lymphatic vessel invasion OR lymphatic invasion OR LVI OR lymph node metastasis OR LNM”. All abstracts that indicated the correlation between LVD, LVI, and LNM in breast cancer, either prospective or retrospective, were chosen for further consideration.

### Inclusion criteria

All studies were required to meet the following criteria: (1) dealt with the patients with primary breast cancer only; (2) published as a full-text research paper, rather than reviews, case reports, meeting abstracts, or animal researches; (3) clearly described the methods and procedures of tissue handling and pathological staining; (4) the determination of LVI presence was assessed by immunohistochemical (IHC) staining instead of hematoxylin and eosin (H&E) staining. When two or more articles reported duplicating data, only the study with the most recent data or the largest dataset was included. Two independent authors followed the inclusion criteria to review the publications. In case of dispute, a third author assessed the study to obtain a consensus.

### Data extraction and quality assessment

Data retrieved from the articles included the first author's name, publication year, country, number of patients, age, type of breast cancer involved, antibody and its dilution, detail data or patient number of LVD/LVI used to determine the correlation between each group. Items intended for extraction were discussed by two authors.

The quality assessment of including studies was based on the criteria of the Newcastle-Ottawa Quality Assessment scale (NOS) (Table S1 in Supplementary file 2) [49]. The scale used a star system to evaluate the study quality, including the aspects of selection, comparability, and exposure. The greater number of stars represented higher-quality studies, which performed better and strictly controlled for potential confounders.

### Statistical analysis

The results showed with detailed data of means and standard deviations, or presented in two by two frequency tables, were used to obtain the Spearman correlation coefficients (*r*) [50]. Because some variables in the original studies were log-transformed before analysis, Spearman's correlation coefficient, instead of Pearson's correlation coefficient, was applied in this study [51]. Before the combination of results, a Fisher's Z transformation was used to convert Spearman's correlation coefficients into an approximately normal distribution (Figure S7 in Supplementary file 1). The individual Fisher's Z values with their corresponding 95% confidence interval (95% CI) were pooled to obtain an overall estimate using STATA 12.0 software. The pooled *Z* value was finally transformed back to *r* value by an inverse Fisher's Z transformation (Figure S7 in Supplementary file 1). Correlation coefficients are not precise but were generally classified as weak, moderate, strong, and excellent. In our study, we assumed < 0.25 to be weak or no correlation, 0.25–0.50 to be moderate, 0.50–0.75 to be strong, > 0.75 to be excellent or perfect [52].

Homogeneity test was performed with *Q* statistic and the *I*^2^ statistic. In this study, *P* < 0.05 or *I*^2^ value > 50% were considered to be statistically significant. A random-effects model or, in the absence of heterogeneity, a fixed-effects model was utilized to combine the Z values. If heterogeneity was noted, a sensitivity analysis was conducted to investigate the influence of each study on the overall estimate by omitting each study in turn. Publication bias was detected by Begg's test and Egger's test.

## CONCLUSIONS

The study describes a triangle relationship of LVD, LVI, and LNM in primary breast cancer. Both LVD and LVI are valuable predict factors of LNM, while LVD moderately correlates with LNM and LVI weakly correlates with LNM. Compared with intratumoral LVD, peritumoral LVD shows a more robust correlation with LVI and LNM, which indicates that peritumoral lymphatic vessel is the main disseminate route for breast tumor cells. It suggests that the patients detected with high LVD or LVI presence, especially with peritumoral high LVD, should take more active treatment to prevent the aggravation and metastasis of primary breast cancer. However, further studies with larger sample sizes should be performed to validate our results.

## SUPPLEMENTARY MATERIALS FIGURES AND TABLES


