# Supplementary material for: The relationship of lymphatic vessel density, lymphovascular invasion, and lymph node metastasis in breast cancer: a systematic review and meta-analysis
Source: Oncotarget. 2016 Dec 1;8(2):2863–73. doi: 10.18632/oncotarget.13752 (PMC5356848; doi:10.18632/oncotarget.13752)
Supplement: Supplementary file 1 [file oncotarget-08-2863-s001.pdf]

The relationship of lymphatic vessel density, lymphovascular invasion, and lymph node metastasis in breast cancer: a systematic review and meta-analysis

Supplementary Materials

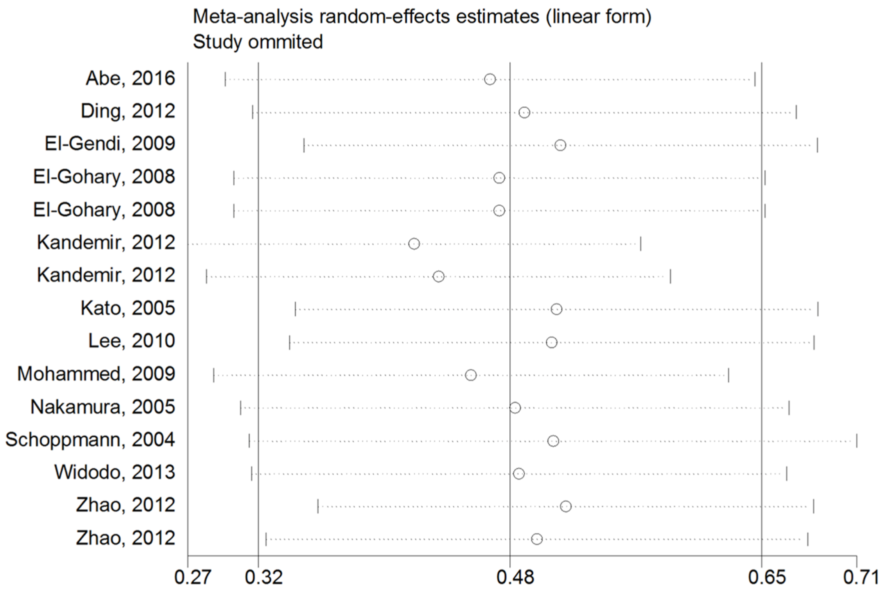

Supplementary Figure S1: Sensitivity analysis of the included studies reporting the relationship between LVD and LVI.

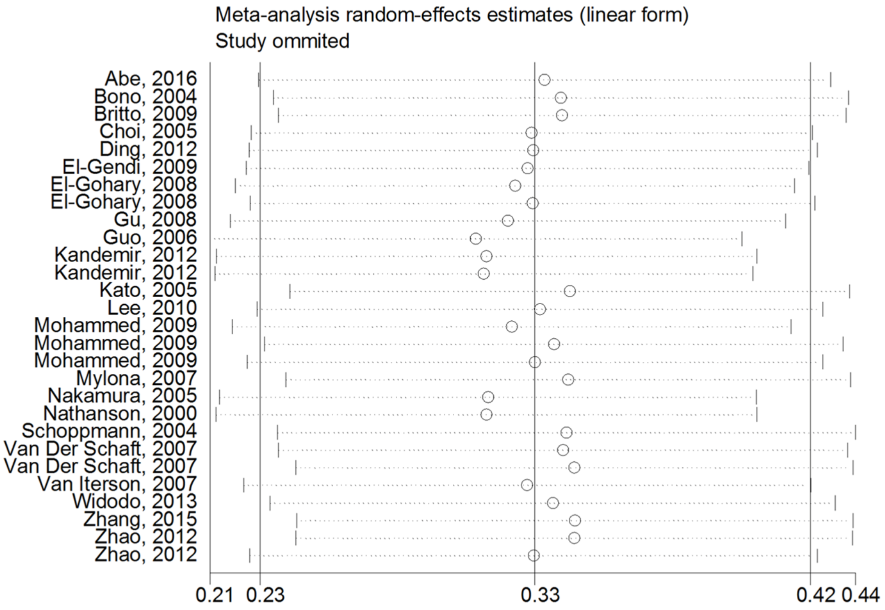

Supplementary Figure S2: Sensitivity analysis of the included studies reporting the relationship between LVD and LNM.

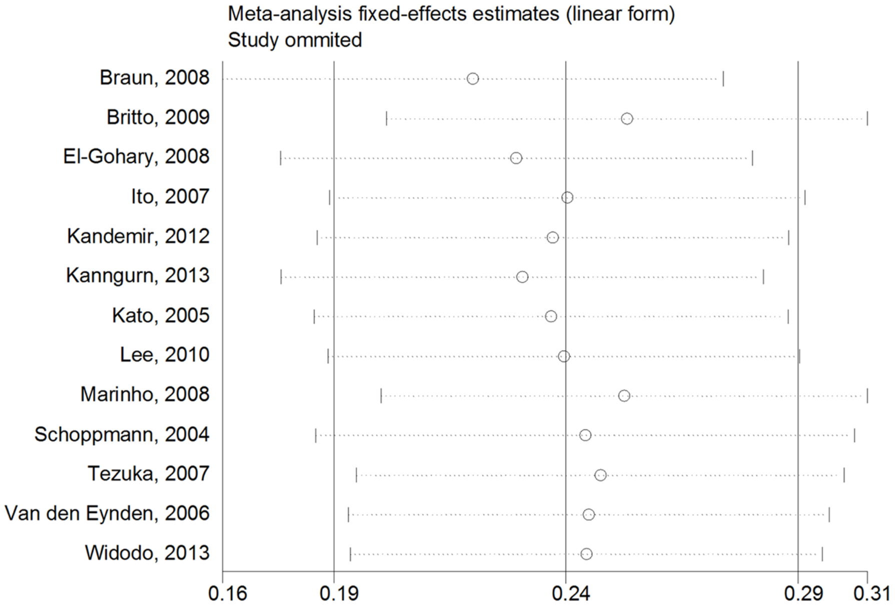

Supplementary Figure S3: Sensitivity analysis of the included studies reporting the relationship between LVI and LNM.

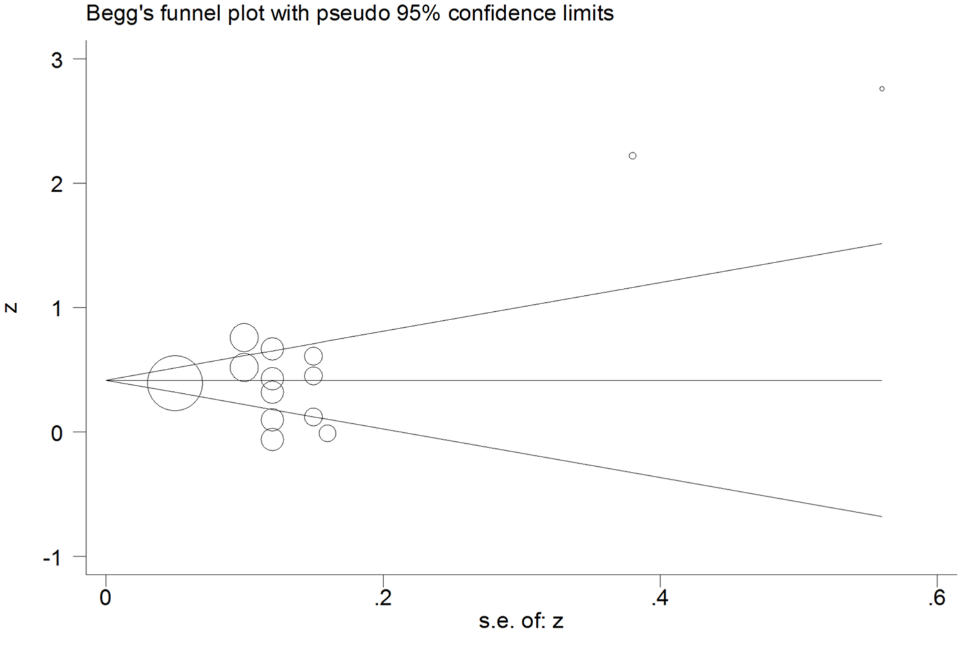

Supplementary Figure S4: Begg's funnel plot of the included studies reporting the relationship between LVD and LVI for publication bias.

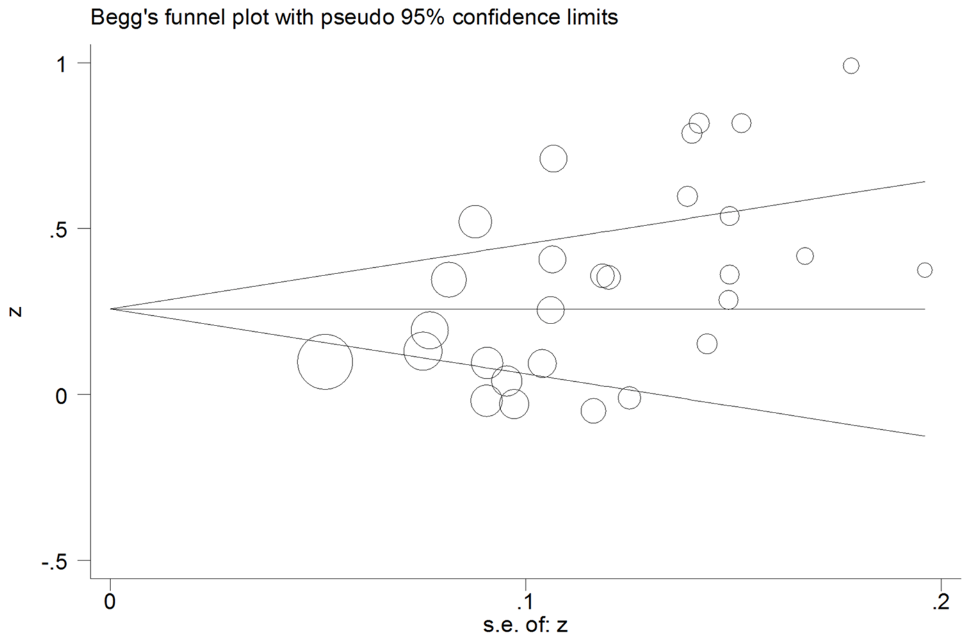

Supplementary Figure S5: Begg's funnel plot of the included studies reporting the relationship between LVD and LNM for publication bias.

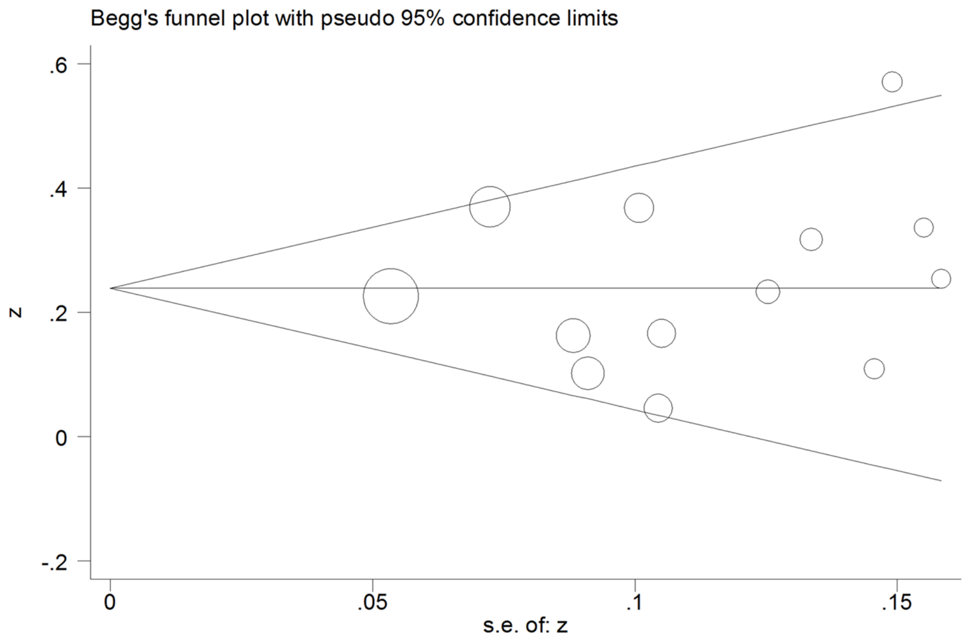

Supplementary Figure S6: Begg's funnel plot of the included studies reporting the relationship between LVI and LNM for publication bias.

[1] $\text{fisher's } z = 0.5 \times \ln\frac{1+r}{1-r}$

[2] $V_z = \frac{1}{n-3}$

[3] $S_e = \sqrt{V_z}$

[4] $\text{Summary } r = \frac{e^{2z}-1}{e^{2z}+1}$ $(z = \text{Summary fisher's } z)$

Supplementary Figure S7: Fisher's Z conversion formulas.

Supplementary Table S1: Assessment of study quality using the Newcastle-Ottawa Scale

Studies	Selection*				Comparability**	Exposure***		
	Is the case definition adequate?	Representativeness of the cases	Selection of controls	Definition of controls	Comparability of cases and controls on the basis of the design or analysis	Ascertain-ment of exposure	Same method of ascertainment for cases and controls	Non-Response Rate
Abe, 2016	✯	✯	✯		✯	✯	✯	
Zhang, 2015	✯	✯	✯	✯	✯	✯	✯	✯
Kanngurn, 2013	✯	✯	✯		✯	✯	✯	
Widodo, 2013	✯	✯			✯	✯		
Ding, 2012	✯	✯	✯		✯ ✯	✯	✯	
Kandemir, 2012	✯	✯	✯	✯	✯	✯	✯	✯
Zhao, 2012	✯	✯	✯		✯	✯	✯	
Lee, 2010	✯	✯	✯	✯	✯	✯	✯	✯
Britto, 2009	✯	✯	✯		✯ ✯	✯	✯	
El-Gendi, 2009	✯	✯	✯	✯	✯	✯	✯	✯
Mohammed, 2009	✯	✯	✯	✯	✯ ✯	✯	✯	✯
Braun, 2008	✯	✯			✯ ✯	✯		
El-Gohary, 2008	✯	✯	✯	✯	✯ ✯	✯	✯	
Gu, 2008	✯	✯	✯		✯ ✯	✯	✯	✯
Marinho, 2008	✯	✯	✯		✯	✯	✯	
Ito, 2007	✯	✯	✯		✯	✯	✯	✯
Mylona, 2007	✯	✯	✯	✯	✯ ✯	✯	✯	
Tezuka, 2007	✯	✯			✯	✯		
van der Schaft, 2007	✯	✯	✯		✯ ✯	✯	✯	✯
van Iterson, 2007	✯	✯	✯	✯	✯	✯	✯	
Guo, 2006	✯	✯	✯		✯	✯	✯	
van den Eynden, 2006	✯	✯	✯	✯	✯	✯	✯	✯
Choi, 2005	✯	✯	✯	✯	✯	✯	✯	
Kato, 2005	✯	✯	✯	✯	✯	✯	✯	
Nakamura, 2005	✯	✯	✯		✯	✯		
Bono, 2004	✯	✯	✯	✯	✯	✯	✯	✯
Schoppmann, 2004	✯	✯	✯		✯ ✯	✯	✯	✯
Nathanson, 2000	✯	✯	✯		✯ ✯	✯	✯	✯

Note: The studies were awarded a maximum of one star for each numbered item within the Selection and Outcome categories. A maximum of two stars was given for Comparability. The maximum total NOS score was nine stars, and the studies with ≧ 6 stars were considered to have relatively higher quality.

* Selection: (1) Is the case definition adequate: a) yes, with independent validation (✯); b) yes, eg record linkage or based on self reports; c) no description. (2) Representativeness of the cases: a) consecutive or obviously representative series of cases (✯); b) potential for selection biases or not stated. (3) Selection of controls: a) community controls (✯); b) hospital controls; c) no description. (4) Definition of controls: a) no history of breast cancer (✯); b) no description of source.

** Comparability: 1) Comparability of cases and controls on the basis of the design or analysis: a) study controls for lymph node status (✯); b) study controls for any additional factor (age) (✯).

*** Exposure: (1) Assessment of exposure: a) secure record (surgical and pathological records) (✯); b) structured interview where blind to case/control status (✯); c) interview not blinded to case/control status; d) written self report or medical record only; e) no description. (2) Same method of ascertainment for cases and controls: a) yes (✯); b) no. (3) Non-Response Rate: a) same rate for both groups (✯); b) non respondents described; c) rate different and no designation.
